# Nuclear Factor Kappa B p65: A Possible Biomarker for Persistent Inflammation in HIV-1 Infection?

**DOI:** 10.7759/cureus.71308

**Published:** 2024-10-12

**Authors:** Sivasubramaniyan Gnanaskandan, Padma Srikanth

**Affiliations:** 1 Microbiology, Sri Ramachandra Institute of Higher Education and Research, Sri Ramachandra Faculty of Allied Health Science, Chennai, IND

**Keywords:** aids, antiretroviral therapies, chronic inflammation, hiv-1 infection, host-immune response, inflammatory marker, msm, nf-kb p65

## Abstract

Low-grade inflammation in people living with HIV (PWH) has become a significant contributor to the development of non-communicable diseases (NCDs) such as heart disease, stroke, and renal dysfunction. Though antiretroviral therapy (ART) has dramatically reduced mortality by limiting the emergence of opportunistic infections, it has not been successful in eliminating the remaining chronic, low-grade inflammation and activation that persists in the infected despite viral suppression and better CD4+ T cell count. Nonetheless, this relatively asymptomatic and subclinical chronic inflammation remains poorly understood and has become a major contributor to mortality in PWH. Another important component involved in this step is the Nuclear Factor kappa B (NF-κB) which is a central transcription factor in the immune system to respond to infection. Specifically, the p65/RELA subunit attaches to the HIV LTR (long terminal repeat) gene and consequently initiates the synthesis of genes related to inflammation and immune reactions. Persistent low-level chronic inflammation contributes to the pathophysiology of metabolic-inflammatory NCDs. Therefore, this review aims to assess the complex contextual function of NF-κB p65 during HIV-1 disease, particularly among individuals on ART who achieve viral suppression. As much as ART has helped to arrest the progression of the virus, immune function, and chronic inflammation have not been reversed in most PWH. It is, therefore, pertinent to know how the NF-κB p65 molecule remains involved in those with persistent immune inflammation concerns to enhance strategies on the same. This review will also discuss the possible variation in NF-κB p65 activity in particular population groups such as MSM (men who have sex with men) to acquire additional information that could potentially enhance the treatment.

## Introduction and background

HIV-1 remains to be a global health threat even today, affecting millions of people across the world [1]. This virus specifically targets CD4+ T cells, the very cells that are essential for our immune defense, gradually weakening the body’s ability to fight against infections. Without treatment, this deterioration can lead to AIDS, a condition that severely compromises the immune system [2]. A key breakthrough in HIV-1 treatment has been the introduction of antiretroviral therapy (ART). Zidovudine (AZT) became the first antiretroviral drug approved for use in the treatment of HIV/AIDS in 1987 when HIV had limited treatment options. However, the introduction and development of highly active antiretroviral therapy (HAART) in 1996 significantly advanced the treatment of HIV-1 [3].

As of 2022, approximately 38 million people globally are living with HIV. The widespread availability of ART has been a game changer, dramatically reducing the number of deaths associated with HIV/AIDS, from 1.7 million in 2004 to about 650,000 in 2022 [4]. In India, an estimated 2.3 million people were living with HIV in 2022; almost 73% of the population had access to ART. The mortality rate in India has also declined as well to about 58,000 per year in 2022 compared to around 200,000 per year in the early 2000s. These statistics corroborate the success of ART in regard to HIV-1 control despite emerging hurdles, especially in pre-ART or those who have never been tested [5].

ART now is given to all HIV-infected individuals irrespective of CD4 counts and HAART encompasses at least three antiretroviral drugs acting at different phases of the HIV-1 life cycle. HAART has been found to be very effective in minimizing viral replication and bringing about viral suppression [6,7]. Today, viral suppression, which is defined by HIV-1 RNA levels below the limit of detection, is feasible for most individuals on ART, resulting in enhanced lifespan and quality of life for those affected by HIV. Nevertheless, it is also important to make the point that though ART can be very beneficial, it actually does not cure the infection. The virus stays in the body, so lifelong treatment is required [8]. Though ART has positively impacted millions by providing them with extra years to live, it has its limitations, especially with regard to chronic health complications. Continued immune activation and inflammation are evident in persons living with HIV even with viral suppression, resulting in the development of conditions such as cardiovascular diseases, neurocognitive disorders, and other non-AIDS-related illnesses [9].

Why there is always an immune activation and inflammation, despite ART? There are various factors influencing the persistence of inflammation and immune activation in individuals on ART and viral suppression: (1) Viral reservoirs: Although ART reduces the plasma HIV RNA to undetectable levels, low-level virus may persist in certain tissues, including lymphoid tissues and the gut [10]. The studies have shown that even in virally suppressed individuals, the presence of HIV DNA in reservoirs can drive immune activation and inflammation via the NF-κB pathway which leads to HIV-associated neurocognitive disorders (HAND) [11-13]; (2) Gut microbial translocation: Dysbiosis of the gut has been studied in HIV-infected individuals on effective ART, where disruptions in the normal gut microbial flora lead to a "leaky gut." This condition allows bacterial products, such as lipopolysaccharides (LPS), to enter the systemic circulation, driving inflammation and immune activation, which in turn increases the risk of noncommunicable diseases [14-16]. Circulating LPS can activate NF-κB p65, which contributes to both immune activation and, potentially, viral replication [17]; (3) Co-infections: Patients with HIV may experience co-infections with other pathogens, including hepatitis viruses, cytomegalovirus (CMV), Epstein-Barr virus or tuberculosis. Each of these co-infections can independently trigger immune activation and inflammation by NF-kB p65 activation, which plays a central role in the inflammatory response [18,19]. These co-infections complicate the separation of immune activation induced by HIV and the sustained immune activation after ART making it difficult to reverse inflammation [20-22]; (4) Immune system dysregulation: HIV-positive individuals experience persistent immune dysfunction, including alteration in T cell subsets, increased numbers of more senile T cells, and activation of monocytes and macrophages continuously. However, these changes may not be completely reversed even with ART leading to sustained immune activation and inflammation [23]. Studies have shown that markers of immune activation, such as CD38 and HLA-DR on T cells, remain elevated in ART-treated individuals compared to uninfected controls [24,25]; (5) Inflammatory cytokine production: Complications associated with chronic HIV infection include increased in pro-inflammatory cytokines, including IL-6, TNF-α, and IL-1β, thus contributing to HIV-associated comorbidities like cardiovascular diseases [26]. These cytokines are usually controlled by the NF-kB p65 pathway, a well-documented promoter of inflammation [27]. Even with ART, inflammation is still present as cytokine levels remain high. The impact of ART became evident that ART failed to restore NF-kB activity as well as cytokine levels, which helps to explain the constant inflammation in people living with HIV [28]. Thus, NF-κB p65 is not only involved in HIV replication or reactivation but is also responsible for the release of inflammatory cytokines by bacterial products, gut dysbiosis, co-infection, and immune dysregulations even ineffective ART.

The NF-kappa B p65 (NF-κB p65) is a nuclear factor protein, that is involved in the modulation of immune responses, inflammation, and cell survival. It is also involved in the immune defense of the body, which allows it to coordinate the response to various infections and other threats through the regulation of genes [29]. Conversely, the NF-κB p65 subunit has an even greater role in the context of HIV-1 infection. Like most viruses, HIV-1 has developed strategies to harness the host cellular machinery for its benefit to improve replication. One such mechanism involves the NF-κB pathway. Activated NF-κB p65 binds to the long terminal repeat (LTR) site of the HIV-1 virus which leads to subsequent transcription of viral genes which is fundamental in the manufacture of new virus particles [30-32].

The interaction between HIV-1 and NF-κB p65 is not limited to viral replication but holds other significance as well. NF-κB p65 activation results in sustained immune stimulation and inflammation even in those patients with suppressive ART and HIV viral load undetectability [33]. Due to the sustained activation of NF-κB p65, inflammation, which HIV is known to induce is perpetuated and therefore results in several complications including cardiovascular diseases, and neurocognitive disorders amongst others [34]. These conditions complicate the overall HIV control and treatment in the long run despite optimal suppression of the virus.

## Review

NF-kappa B, an overview

The nuclear factor-kappa B (NF-κB) family is a critical group of transcription factors involved in numerous cellular processes, including immune responses, inflammation, apoptosis, and cell survival. It was first identified in 1986 by David Baltimore and his coworkers as a nuclear factor that binds the enhancer region of the kappa light chain of immunoglobulin in B cells. Though it was initially linked with B cells, it is now obvious that NF-κB exists as a repressed form, cytoplasmic, in almost all cell types and has a degree of conservation, which goes from Drosophila to humans, whereas, upon activation, it translocates into the nucleus, constituting an essential factor for the regulation of the expression of more than 300 genes whose products are associated with immunity and growth as well as inflammation. There exist five members of the NF-κB family that have been identified: NF-κB1 (p50/p105), NF-κB2 (p52/p100), RelA (p65), RelB, and c-Rel. There are two major pathways to activate NF-κB: the canonical and non-canonical. Upon activation, NF-κB1 and NF-κB2 are processed into their active forms, p50 and p52, respectively, before entering the nucleus [35,36].

The NF-κB dimer of quiescent cells mainly consists of p50 and RelA subunits and is inactive in the cytoplasm as it has associating inhibitory proteins IκBα, IκBβ, IκBγ, p105, and p100, of which IκBα is the most predominant. This is the inactive NF-κB/ IκB complex that will be activated upon phosphorylation of the two conserved S residues in the N-terminal domain of the IκB proteins. Thus, in response to external stimuli, the polyubiquitinated IκB proteins are catalyzed by SCF-β-TrCP; hence, they are marked for rapid degradation by the 26S proteasome. With the degradation of the IκB, NF-κB is released and free to move inside the nucleus and proceed with gene expression [37].

The transcription factor NF-κB is a DNA-binding protein complex that regulates the transcription of genes. It was discovered 15 years ago; a significant discovery in 1986 identified NF-κB as a factor controlling the production of the kappa variety of the immunoglobulin light chain, one key component of antibodies. Then called for its position in controlling kappa light chain expression in B cells, NF-κB was first identified there. Since then, it has been established that NF-κB participates in inflammation, cell growth, and programmed cell death (apoptosis) and plays a role in diseases such as cancer, arthritis, and asthma. Such proteins are among the most important constituents of a family of transcription factors composed of dimers of Rel proteins, which share a common Rel homology domain (RHD). NF-κB plays a critical role in regulating both immune response and inflammation and is involved in tumorigenesis. Once activated, it leads to the near complete degradation of IκB proteins, enabling NF-κB to enter the nucleus and bind to specific DNA sequences known as κB elements. This binding activates the transcription of more than 400 genes involved in immune function, growth regulation, inflammation, cancer development, and apoptosis [37,38].

Families of NF-κB

**Figure 1:**
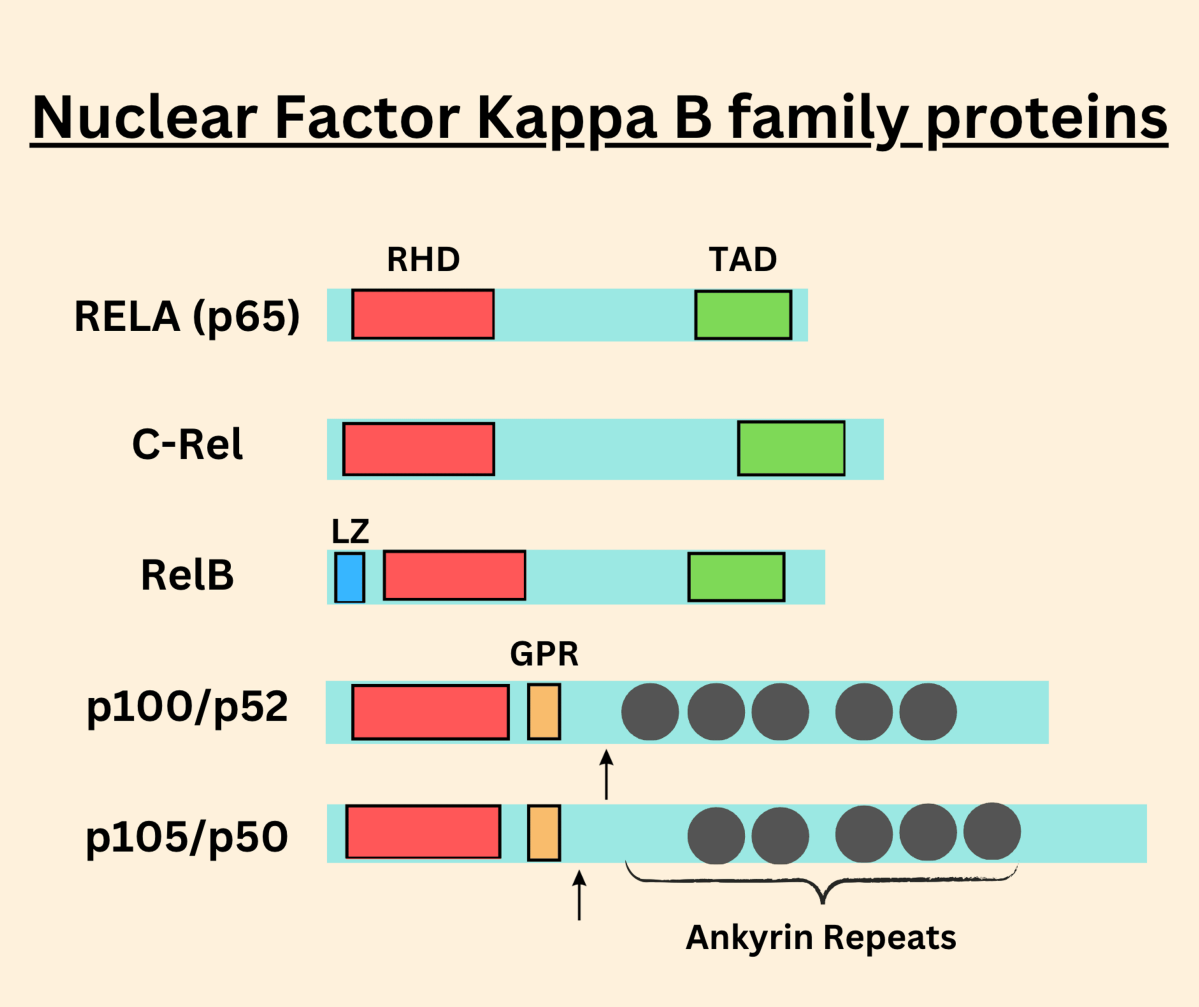
NF-κB proteins. All five mammalian Rel-related proteins—p65 (RelA), c-Rel, RelB, p100/p52, and p105/p50—share a Rel Homology Domain (RHD) – red box, which allows them to dimerize and bind to DNA. Among them, only RelA, c-Rel, and RelB feature a C-terminal Transactivation Domain (TAD) – green box, crucial for activating gene transcription. p100 and p105 have ankyrin repeats (indicated by dark gray circle), which help them bind NF-κB dimers and keep them in the cytoplasm. To generate the active p52 and p50 subunits, p100 and p105 undergo ubiquitin-dependent processing, which removes their C-terminal portions (shown by the arrow at the cleavage point). Glycine-rich region (GRR), and leucine zipper (LZ). Figure created by authors using Canva (paid version).

There are five transcription factors in the NF-κB family: p50, p52, RelA (p65), c-Rel, and Rel-B. These factors contain a common 300-amino-acid N-terminal region called the Rel homology domain, or RHD, which enables them to form both homodimers and heterodimers that can bind to the specific 9-10 base pair specific DNA sequences known as κB sites in the promoter and enhancer regions of genes, thus modulating gene expression. However, only RelA, c-Rel, and RelB possess a C-terminal Transactivation Domain (TAD) that is required for the transactivation of target gene expression (Figure 1). TAD provides these proteins with a role in acting as transcriptional activators in regulating many genes that encode proteins associated with the immune response, inflammatory environment, and other cellular functions. As shown above, p50 and p52 lack TAD, and cannot initiate transcription independently, except dimerized with other members of the NF-κB family that possess TAD. Conversely, beyond this dimer, they behave as more of a transcriptional repressor [39].

Interestingly, p100 and p105, precursors to p52 and p50, respectively, have another characteristic: ankyrin repeats. The ankyrin repeats, as diagrammed-dark grey circle-are important in keeping the NF-κB dimers latent through association with them and sequestered into the cytoplasm (Figure 1), so the dimer does not become translocated to the nucleus until the signal to do so is received. With ubiquitin-dependent proteolytic processing, the C-terminal domain of p100 or p105 can be removed to generate the active NF-κB subunits, p50 and p52. In this process, the free active p50 or p52 will also result from stripping off the remaining C-terminal portion of the molecule. The point of the arrow indicates the location where the C-terminal end of the protein is actually cleaved off to release these proteins for signaling function in NF-κB [39,40]. Other functional domains besides the ankyrin repeats are present in these proteins: These are the glycine-rich region and the leucine zipper, both essential to provide structural stability and function for these proteins. The glycine-rich region specific to a number of NF-κB family members confers some flexibility, whereas the leucine zipper is associated with contacts between diverse protein domains [39].

IκB kinase

The activation of various stimuli on a cell triggers a signal transduction pathway that ultimately points to the induction of specific IκB kinases, or IKKs. IKKs are critical regulators of the NF-κB signaling pathway critically important for innate immunity and inflammation and for protection against cell death.

The three major subunits of the IKK complex are IKKα or IKK1, IKKβ or IKK2, and IKKγ, also known as NF-κB essential modulator or NEMO. Though the two catalytic subunits, IKKα and IKKβ, have an overall identity of 52% and a similarity of 65% at their catalytic domains, IKKγ/NEMO is a regulatory subunit which is not homogenous concerning its relationship as the catalytic subunits. In vitro, both IKKα and IKKβ exhibit identical substrate specificity. They phosphorylate specific serine residues in the N-terminal regulatory domains of the IκB proteins. Nonetheless, IKKβ is more efficient in IκB phosphorylation than IKKα. This complex is activated by a vast array of stimuli that also activate NF-κB, such as microbial, fungal, and viral products, as well as pro-inflammatory cytokines. Thus, the induction of NF-κB signaling is associated with the activation of IKK.

When phosphorylated by IKK, the IκB proteins are targeted for polyubiquitination by a ubiquitin ligase belonging to the SCF Skp-1/Cul/F-box family. After phosphorylation, the IκB proteins are recognized by the WD-repeat and F-box protein, β-TrCP. After ubiquitination, the IκB proteins are rapidly degraded in the proteasome. This degradation frees NF-κB so that it translocates into the nucleus where it binds to DNA, and activates transcription of genes. Apart from controlling the phosphorylation of IκB proteins, the IKK complex also directly participates in a phosphorylation event in the case of the p100 protein that leads to the activation of dimers p52 which may add further to NF-κB signaling and control of gene expression [39].

NF-κB pathway

The NF-κB pathway actually is divided into two main branches to induce NF-κB activation, the canonical (or classical) pathway and the non-canonical (or alternative) pathway. Both pathways possess a common controlling step in the activation of IKK complex, which later contains catalytic kinase subunits IKKα and IKKβ, whereas the regulatory scaffold NEMO is also known as IKKγ.

In both mechanisms, IKK phosphorylates the NF-κB dimers that cause the proteasomal degradation of the inhibitor IκB. This is to allow the efflux of NF-κB complex to the nucleus to initiate the transcriptional activation of target genes, such as the IκBα gene as explained by Strickland and Ghosh, 2006. IκBα binds with the NF-κB subunits in the cytoplasm to terminate the transcriptional function in case the transcription factor does not maintain a constitutive activation state.

Canonical/Classical Pathway

In the classical pathway, the binding of a ligand to the cell surface receptor, such as the toll-like receptor family, recruits adaptor proteins, including TRAF (TNF receptor-associated factor), to the cytoplasmic domain of the receptor. The adaptor proteins recruit the IKK complex, which leads to the phosphorylation and degradation of the IκB inhibitor, thereby activating dimers of NF-κB consisting of the components RelA, c-Rel, RelB, and p50. Other effects attributed to the canonical pathway include activation of innate immunity, promotion of inflammation, and prevention of apoptosis [37].

Non-canonical/Alternative Pathway

Activation of p100/Rel-B complexes is mainly mediated by the non-canonical pathway; however, this pathway is necessary for the development of lymphoid organs, which constitute part of the environment necessary for B and T lymphocyte maturation. This type of pathway can also be elicited by BAFF (B-cell activating factor) and other factors through a distinct IKK complex that contains two catalytic subunits of IKKα and lacks NEMO. The non-canonical pathway reports to activate NF-κB-inducing kinase, NIK with activation by ligand. The latter is a known enhancer of phosphorylation and thus activation of the IKKα complex, which responds by inducing its own phosphorylation of p100. This is followed by the processing and release of active p52/Rel-B heterodimers. Whether the processing of p105, receiving constant cleavage to produce p50, is itself inducible is controversial. In contrast, genetic deletions of elements of the non-canonical pathway, including those for NIK and IKKα, have implicated it in several essential functions including B cell maturation, development of secondary lymphoid organs, high-affinity antibody production, and oncogenic chemokine biosynthesis [37,41].

Activation of NF-κB p65 in immune response, inflammation, and cellular processes

In general, if the body is threatened by foreign antigens, NF-κB is responsible for summoning adaptive and innate immune cells like macrophages, to initiate inflammation [42]. Inflammation is typically a short-term process that decreases as soon as threats are overcome to prevent significant collateral damage to the body. For example, signals that activate NF-κB can stimulate the making of interferons and stimulating genes that give protection against bacterial and viral pathogens [43]. However, when the immune system becomes overactive, it results in acute or chronic inflammation which causes tissue damage as seen in inflammatory diseases. Also, NF-κB helps the immune system by enhancing the production and development of immune cells and the regulation of death and survival signaling pathways through the regulation of pro-apoptotic and anti-apoptotic factors.

**Figure 2:**
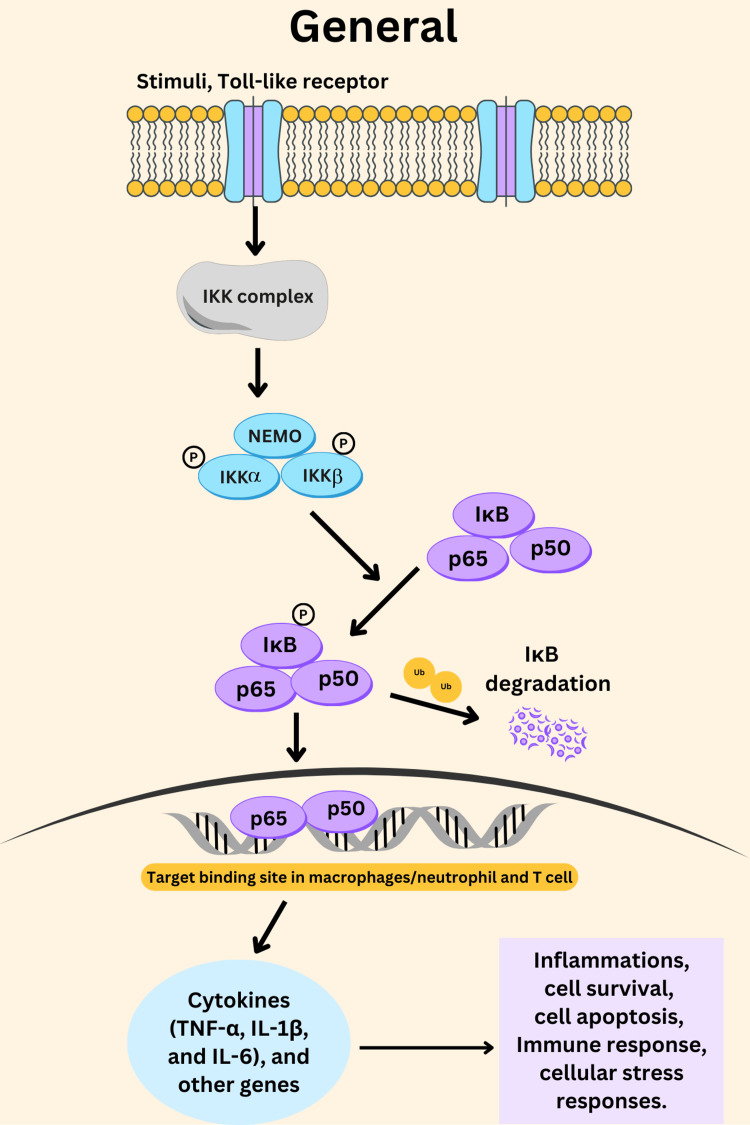
Activation of NF-κB p65 by stimuli (leads to cytokines production and inflammation). Figure created by authors using CANVA (paid version).

NF-κB p65 is involved in the control of immune and inflammatory responses. It plays a crucial role within the innate immune system which is the body’s initial response to numerous illnesses. When microbial products bind to pattern recognition receptors (PRRs), such as toll-like receptors (TLRs), the IKK complex, which consists of IKKα, IKKβ, and IKKγ (also called NEMO), is activated. This complex plays a critical role in the NF-κB signaling pathway by phosphorylating the inhibitor of NF-κB, known as IκB (typically IκBα). Once IκB is phosphorylated by IKKβ, it undergoes ubiquitination, marking it for degradation by the proteasome. As IκB is degraded, the NF-κB dimer, consisting of p65 (RELA) and p50, is released from its inhibitory complex. Freed from IκB, the p65/p50 dimer translocates into the nucleus, where it binds to specific DNA sequences called κB sites in the promoter regions of target genes (macrophages, neutrophils, or T cells). This binding initiates the transcription of various immune and inflammatory response genes, such as TNF-α, IL-1β, and IL-6, which are essential for the body’s defense mechanisms (Figure 2). The accurate regulation of this pathway is crucial, as improper activation can lead to excessive inflammation and contribute to various chronic diseases [40,44,45].

Thus NF-κB p65 is involved in inflammation not only in immediate immune reactions but also in other processes such as cell survival and apoptosis, chronic inflammatory diseases, cancer progression, cellular stress responses, metabolic regulation, and cardiovascular diseases [46-50]. This transcription factor is continually activated in different forms of inflammation, such as inflammatory bowel disease, rheumatoid arthritis, and certain types of cancer [46,51]. The constant activation of NF-κB p65 results in the constant generation of pro-inflammatory cytokines and chemokines which result in prolonged inflammation. chronic inflammation, in turn, can lead to tissue damage and disease progression [42].

Besides its function in immunity and inflammation, NF-κB p65 participates in cell survival and apoptosis. Thus, NF-κB p65 can act as either a survival factor or a factor that initiates cell death. For example, in relation to some stress signals, the NF-κB p65 can activate the genes that promote anti-apoptosis, including B-cell lymphoma 2 (Bcl-2) and B-cell lymphoma extra-large (Bcl-xL) owned by the cell [52]. In addition, NF-κB p65 is implicated in the processes of cell cycle and differentiation. It is important for the maturation and operation of numerous host defense cells such as T-cells, B-cells, and macrophages [40,42]. By modulating the expression of genes involved in cell cycle regulation and differentiation, NF-κB p65 influences the development of immune cells and their ability to respond to infections.

Activation of NF-κB p65 in HIV-1 replication

NF-κB p65 plays an important role in the life cycle of HIV-1, and understanding how HIV-1 activates and regulates this transcription factor is key to unraveling the complexities of HIV-1 pathogenesis. The activation of NF-κB p65 by HIV-1 is primarily initiated through the interaction of the virus with host cell receptors. When HIV-1 binds to CD4 receptors and co-receptors such as CCR5 or CXCR4 on the surface of target cells, it triggers a cascade of intracellular signaling events.

**Figure 3:**
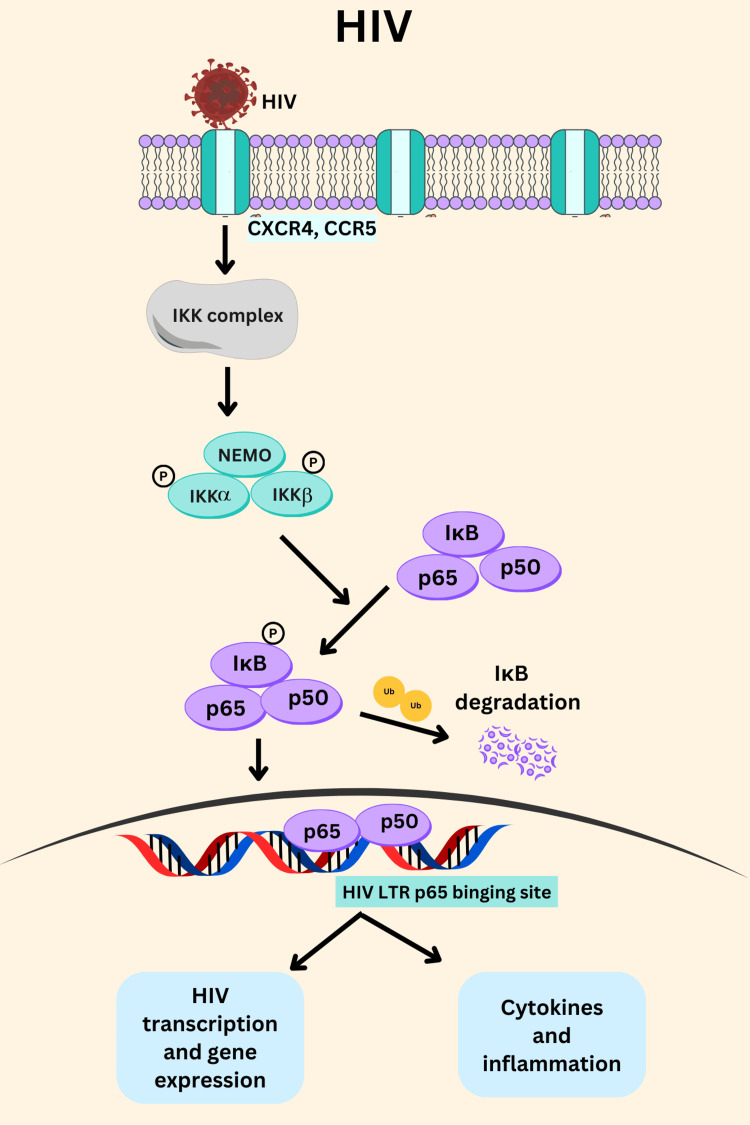
Activation of NF-κB p65 in HIV infection which leads to transcription, gene expression and cytokines productions. Figure created by authors using CANVA (paid version).

Upon binding the IKK complex (IKKα, IKKβ, and NEMO) is activated. Followed by the phosphorylation of IκB by IKKβ, it undergoes ubiquitination in the cytoplasm, marking it for degradation by the proteasome [53,54]. As IκB is degraded, the NF-κB dimer p65/p50 is released from its inhibitory complex and translocated into the NF-κB p65 binding site which is present in the long terminal repeat (LTR) region of the HIV [55], which leads to the production of viral RNA, which is then translated into viral protein and packaged into new virions (Figure 3).

However, the role of NF-κB p65 extends beyond active replication. It is also involved in the maintenance of HIV-1 latency, a state in which the virus remains dormant within infected cells, evading the host immune response and antiretroviral therapy [56,57]. The persistence of HIV-1 in latent reservoirs is a major obstacle to curing the infection. NF-κB p65 contributes to the reactivation of latent HIV-1 by responding to various stimuli, such as pro-inflammatory cytokines or other stress signals, that induce its activation. When NF-κB p65 is activated in latently infected cells, it can bind to the LTR and initiate the transcription of the viral genome, leading to the re-emergence of active viral replication [58].

Factors influencing NF-κB p65 activation lead to HIV-1 replication

HIV interacts with cellular and viral elements leading to the replication of viruses. It has the following mechanisms for activating NF-κB p65, which play a significant role in the process of viral replication, immune response, and inflammation. The major ways NF-κB p65 is activated in HIV are as follows.

HIV Virus Proteins

Tat protein: The HIV-1 Tat protein directly activates NF-κB p65, for that it interacts with the cellular proteins and signaling pathways, which finally leads to the degradation of IκB proteins. This process eventually causes the translocation of NF-κB p65 from the cytoplasm into the nucleus, where it enhances the transcription of HIV by binding to the viral long terminal repeat promoter region [59].

Nef protein: The HIV-1 Nef protein also could associate and activate NF-κB p65 by interacting with other signaling partners, such as TRAFs, thus promoting the activation of the canonical NF-κB pathway supporting viral replication and functioning in immune activation [60].

Glycoprotein 120: The mechanism of HIV replication through activation of NF-κB via a gp120-induced caspase-8-dependent pathway. In this pathway, HIV-1 envelope protein gp120 binds to the cell surface, initiating a signaling cascade that culminates in the activation of caspase-8, an enzyme that has largely been implicated in apoptosis. However, in this instance, caspase-8 plays a role in NF-κB activation, leading to its translocation into the nucleus where it regulates gene expression pertinent to inflammation and viral replication. Such a mechanism emphasizes the dual role of caspase-8 in cell death and immune activation, sustaining the persistence and replication of HIV within host cells [61].

Activated by Cytokines

Pro-inflammatory cytokines, activated by HIV infection, include TNF-α, IL-1β, and IL-6. The pro-inflammatory cytokines activate the canonical pathway of NF-κB through phosphorylation, followed by IκB degradation, where activation through TNFR (Tumour Necrosis Factor Receptor) enables NF-κB p65 to enter into the nucleus. Thus, NF-κB p65 leads to the transcriptional induction of inflammatory genes and HIV LTR [62].

Oxidative Stress

HIV infection leads to preferential enhancement of the oxidative stress that immune cells experience, mainly through an overproduction of ROS (Reactive Oxygen Species). The ROS are also described as signaling molecules or second messengers that trigger various intracellular pathways. ROS leads to IKK activation that governs NF-κB-catalysed transcriptional activation of the 5' LTR region of HIV to favor viral gene transcription and enhanced viral replication [63].

Co-infections

Co-infection with other pathogens, including cytomegalovirus (CMV) and tuberculosis (TB), can cause activation of NF-κB in an individual infected with HIV. These co-infections lead to a chronic inflammatory environment that keeps the NF-κB signaling pathway actively turned on within cells infected with HIV, thus perpetuating the inflammation and viral transcription and promoting the ongoing replication of HIV [64,65].

Toll-like Receptor (TLR) Signaling

These interactions can include an interaction with the Toll-like receptors, especially TLR4 on macrophages and dendritic cells, which activates the NF-κB pathway. Following TLR activation, adaptor proteins such as MyD88 are recruited, and the activation of the IKK complex leads to the translocation of NF-κB p65 in the nucleus, while heightened replication and immune activation of HIV [66].

Endotoxin and Microbial Translocation

Generally, it is the compromised gut barrier function by the HIV infection, which subsequently leads to the activation of translocation of microbes and consequently elevated levels of endotoxins within the blood flow. The LPS (lipopolysaccharide), which is part of the endotoxins, activates NF-κB through TLR4 and triggers long-lasting inflammation among HIV-infected patients by further activation of the NF-κB p65 [67,68].

Factors influencing NF-κB p65 activation in virally suppressed on antiretroviral therapy HIV-1 infection

The fact that NF-κB p65 might be activated in PWH even under ART underscores persistent immune activation and inflammation triggered by various reasons that may lead to long-term complications despite undetectable viral loads. Below, the major mechanisms of NF-κB p65 activation in HIV patients on ART are described

Residual Immune Activation and Inflammation

There is residual low-level immune activation even in the setting of ART. There is considerable evidence that chronic activation may be driven by inflammation of the immune system. This low-level activation has been proposed to arise from cytokines such as TNF-α, IL-6, and IL-1β, which all drive the canonical NF-κB pathway. These cytokines interact with their relevant receptors. This leads to a cascade that begins with the phosphorylation and degradation of IκB and leads to NF-κB p65 translocation into the nucleus where it promotes the expression of inflammatory genes [27,42].

Gut Microbial Translocation

ART is unable to fully restore the integrity of the gut barrier, and the integrity of the gut barrier remains compromised in most virally suppressed patients. This predisposes to the leakage into the bloodstream of bacterial products such as LPS. The process has been termed microbial translocation. LPS activates TLR4 on the immune cells, which has an effect on the NF-κB p65 pathway. The end outcome is chronic inflammation that even under the best ARTs continues to damage the tissue and cause immune activation [67,68].

Co-infections and Inflammation

Continued co-infections, for example, with herpes viruses such as cytomegalovirus or mycobacterium tuberculosis, together with opportunistic infections may perpetually drive immune reactions in HIV-infected individuals undergoing ART. These co-infections can activate NF-κB via several pathways of signaling, causing inflammation to continue unabated even in the presence of suppressed viruses by antiretroviral therapy. Many of these responses commonly cause the transactivation of the NF-κB p65 subunit and promote a pro-inflammatory environment [69,70].

Low-Level HIV Transcription in Reservoirs

ART reduces active HIV replication, but it does not eradicate latent HIV reservoirs in tissues including lymphoid organs and gut-associated lymphoid tissue, and also in resting CD4+ T cells. Low-level, episodic expression of HIV genes from these reservoirs may be capable of stimulating the immune response. The HIV LTR promoter region contains NF-κB binding sites. Even low-level HIV gene expression leads to NF-κB p65 activation [57].

Oxidative Stress

ART, especially for longer durations, has been associated with mitochondrial dysfunction and oxidative stress. Elevated ROS levels can activate NF-κB via its canonical pathway. ROS increases the activity of IKK, which leads to IκB protein phosphorylation and degradation, thus clearing the pathway for NF-κB p65 translocation into the nucleus. This is one of the factors causing a chronic pro-inflammatory environment despite repression of viral replication [71].

TLR Activation by Microbial Products

Microbial byproducts from translocation across the gut and from other sources continue to activate TLRs expressed by the immune cells. TLR activation, such as TLR4, leads to downstream signaling with the activation of NF-κB. The pathway is active in the virally suppressed due to the continued presence of microbial byproducts, with inflammation mediated by NF-κB p65 [66].

ART-Induced Toxicity

Some ART drugs have been associated with low levels of toxicity, possibly through inflammatory responses. Such responses have been put forward to be a product of NF-κB p65 activation through mechanisms of cellular stress, which drives inflammation and immune activation in virally suppressed persons [72].

Inflammatory markers in HIV-1 infection

**Figure 4:**
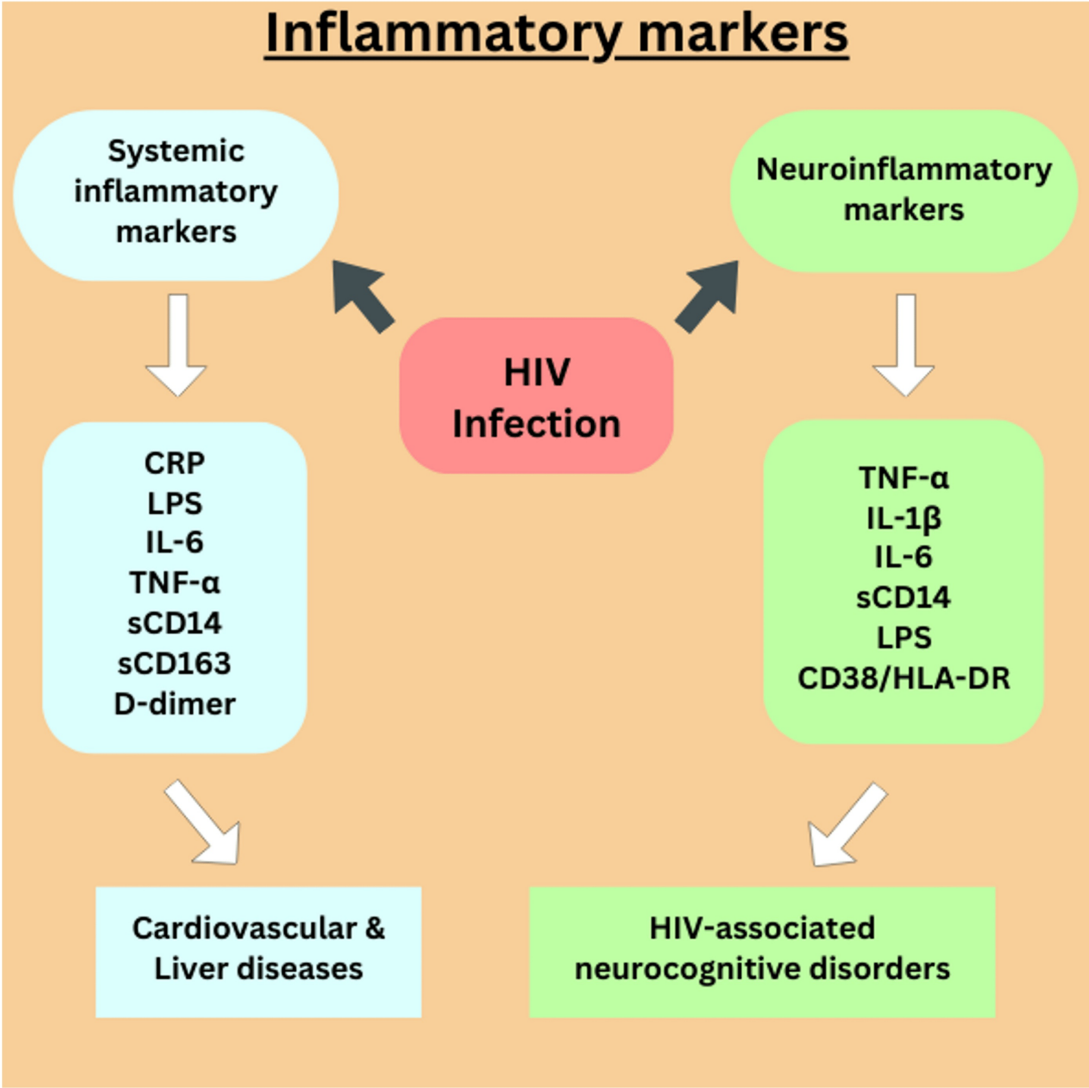
Inflammatory markers in HIV-1 infection. Inflammatory markers and disease progressions: The markers C-reactive protein (CRP), lipopolysaccharide (LPS), interleukin-6 (IL-6), tumor necrosis factor-alpha (TNF-α), soluble CD14 (sCD14), soluble CD163 (sCD163), and D-dimer which is responsible for systemic inflammation and the markers Tumor Necrosis Factor-alpha (TNF-α), Interleukin-1 beta (IL-1β), Interleukin-6 (IL-6), Soluble Cluster of Differentiation 14 (sCD14), lipopolysaccharide (LPS), and Cluster of Differentiation 38 / Human Leukocyte Antigen – DR isotype (CD38/HLA-DR) are the markers associated with HIV-associated neurocognitive disorders (HAND). Image created by authors using Canva (paid version).

In HIV infection, both systemic and neuroinflammatory markers are involved in chronic inflammation, even in individuals on ART. Systemic inflammatory markers such as C-reactive protein (CRP), lipopolysaccharide (LPS), interleukin-6 (IL-6), tumor necrosis factor-alpha (TNF-α), soluble CD14 (sCD14), soluble CD163 (sCD163), and D-dimer are linked to cardiovascular and liver diseases (Figure 4). Fasting triglycerides and CRP, synthesized by the liver in response to IL-6, are associated with cardiovascular disease in people living with HIV. Elevated LPS levels, arising from microbial translocation due to gut barrier dysfunction, activate the immune system, as evidenced by increased sCD14 and sCD163, contributing to cardiovascular and liver complications [73,74]. Neuroinflammatory markers, including TNF-α, IL-1β, IL-6, sCD14, LPS, and upregulated CD38/HLA-DR on T cells, are directly linked to HIV-associated neurocognitive disorders (HAND). These markers compromise the integrity of the blood-brain barrier and facilitate the infiltration of inflammatory cells into the central nervous system (CNS), leading to cognitive impairment ranging from mild neurocognitive dysfunction to HIV-associated dementia. Altogether, both systemic and neuroinflammatory markers reflect the dual impact of chronic inflammation in HIV, affecting both the body and brain [75].

Why NF-κB p65 is a possible biomarker in HIV-1 infection?

While the aforementioned markers are well-established in reflecting systemic inflammation, NF-κB p65 stands out due to its central role in driving immune activation and inflammation at a cellular level in HIV infection. NF-κB is a transcription factor that regulates the expression of many pro-inflammatory cytokines, including IL-6, TNF-α, and IL-1β. Its sustained activation during HIV infection promotes chronic inflammation, contributing to immune dysfunction.

NF-κB is more than just a marker; it is a master regulator of the inflammatory response. Unlike markers such as CRP or sCD14, which are downstream indicators of inflammation, NF-κB directly initiates and amplifies the inflammatory response. This positions it as a key driver of immune activation, which persists even during ART, making it a critical biomarker for understanding chronic inflammation in people living with HIV.

One of the most significant consequences of NF-κB p65 activation in HIV-1 infection is its contribution to chronic inflammation, a hallmark of HIV-1 pathogenesis even in the era of effective antiretroviral therapy (ART) [33,76]. This ongoing inflammation is a critical factor contributing to various non-communicable diseases (NCDs) in people living with HIV (PWH) [77].

Several studies have shown that NF-κB p65 is persistently activated in HIV-1-infected individuals, which leads to the production of inflammatory cytokines such as TNF-α, IL-6, and IL-1β, even in suppressed viral replication [27,42]. This persistent activation may be driven by low-level viral replication, microbial translocation, and co-infections, all of which can continuously stimulate NF-κB pathways even under effective ART [78].

Moreover, the activation of NF-κB p65 by HIV-1 is not limited to direct viral effects; it also involves indirect mechanisms, such as the release of microbial products from the gut due to increased intestinal permeability in HIV-1 infection. These microbial products can further activate NF-κB p65, amplifying the inflammatory response and contributing to the systemic inflammation observed in people living with HIV [79].

Interestingly, various cell culture based studies suggest that NF-κB p65 inhibitors could be potential therapeutic agents in reducing inflammation and associated NCDs in HIV-1-infected individuals. By specifically targeting this pathway, it may be possible to decrease the production of pro-inflammatory cytokines and mitigate the chronic inflammatory state [80-82]. However, the challenge remains in balancing the suppression of inflammation without compromising the necessary immune responses that protect against opportunistic infections and other pathogens.

MSM (Men Who Have Sex With Men): Sexual Practices and Inflammation

Inflammation in MSM living with HIV-1 is a complex and multifactorial issue. Despite effective viral suppression through antiretroviral therapy (ART), MSM with HIV-1 continues to experience chronic low-grade inflammation, which is associated with an increased risk of comorbidities and metabolic complications. This persistent inflammation is believed to be driven by several factors, including residual viral replication and microbial translocation, both of which lead to continuous immune system activation even in the absence of active viral replication [83-85].

Could NF-κB p65 play a role in inflammation among MSM living with HIV-1? A study from Chicago suggests that sexual practices specific to MSM populations may contribute to increased levels of inflammatory markers, such as TNF-α [86]. Additionally, two other small studies have highlighted an increase in potential cardiovascular biomarkers and risk factors among the MSM population [87,88]. In one of our cohort studies, currently under review, we compared the levels of NF-κB p65 and highly sensitive C-reactive protein (HS-CRP) in virally suppressed people with HIV (PWH), both MSM (men who have sex with men) and heterosexual women, to those in HIV-negative MSM and women. Our findings revealed that NF-κB p65 levels were elevated in PWH, regardless of whether they were MSM or women along with a trend to increase in HS-CRP. This suggests that the increase in NF-κB p65 is associated more with HIV status than with MSM status.

MSM populations, who often engage in condomless receptive anal intercourse, have been found to be increased in levels of pro-inflammatory cytokines such as interferon γ (IFN γ) and IL-17. These elevated cytokine levels can lead to microtrauma and increased permeability of the rectal mucosa [89,90]. The physical trauma associated with these sexual practices may also contribute to ongoing local and systemic inflammation. Consequently, even when HIV-1 is well-controlled by ART, these factors can sustain an inflammatory environment, leading to health complications that are not fully mitigated by ART alone [85]. It remains unclear whether the elevated inflammation observed in MSM is primarily due to the activation of NF-κB p65 by HIV-1 infection or the associated sexual practices. Since NF-κB p65 is one of the proteins responsible for activating pro-inflammatory cytokines, could inflammation be controlled by treating with NF-κB p65 inhibitors, thereby preventing non-communicable diseases in PWH MSM populations?

Further understanding of the involvement of NF-κB p65 in inflammation in MSM with HIV-1 and whether the specific sexual practices affecting MSM populations contribute to raised inflammation levels is limited. Nevertheless, it is still unknown whether increased inflammation levels are associated only with HIV-1 infection with other practices characteristic of MSM, or with both. MSM populations, who frequently practice receptive anal intercourse, receive injections of semen deep into the gastrointestinal tract, which can produce microtraumas and elevated rectal mucosal permeability [91,92].

The permeability may lead to the passage of microbial products like lipopolysaccharides (LPS) and subsequent activation of the immune system through the NF-κB pathway [93]. This activation of NF-κB p65 increases the formation of pro-inflammatory cytokines leading to continuous inflammation [94,95]. Nevertheless, these observations do not tell us how much of this inflammation is contributed directly by the HIV-1 infection as opposed to the MSM’s sexual practices.

Despite that, it is still hard to make clear conclusions regarding the role of HIV-1 as a cause of certain pathologies, especially with regard to sexual practices since it is very difficult to separate the impact of the virus on the body from the impact of these specific practices. However, MSM with HIV-1 might also have chronic inflammation resulting from co-infections like STIs since the MSM group is more susceptible to getting those infections [96,97]. These co-infections may also trigger the nuclear factor-kappa B p65, thus increasing inflammation. Various study on people living with HIV-1 shows that inflammation levels due to STIs are high [98-100], therefore the importance of designing interventions to help manage chronic inflammation in virally suppressed PWH populations is needed.

New Therapy Strategy Approach

In the ongoing pursuit of an HIV cure, two main therapeutic strategies are being explored: The two methods developed are called “block and lock” and “shock and kill.” With the block and lock technique, the goal is to stop the virus at the transcript level, which in effect means that it becomes dormant [101,102]. In this way, researchers aim to achieve a status where the virus remains dormant and there is no chance of it flaring up if the patient decides to stop using ART [103].

On the other hand, the shock and kill approach is a strategy of restoring the dormant HIV using latency reversal agents (LRAs), which are capable of either provoking the immune system or the virus to eliminate the infected cells. Of these LRAs, the NF activation stimulants such as PKC agonists (PKCa) have proven effective in reactivating HIV latency in both experimental and clinical conditions. Ingenol mebutate (PEP005) and SMAC (Second Mitochondrial Activator of Caspases) mimetics are some of the recent compounds discovered to show that the NF-κB pathway can be a potential clinical target. Notably, PKCa has demonstrated flexibility in its ability to reactivate memory CD4+ T cells in all types of memory T cells, something that is not true of other LRAs [104].

However, regarding the biological range of effects where both “shock and kill” and “block and lock” approaches aim at reactivation of latent HIV and destruction of infected cells, there remains uncertain about their effects on the chronic low-grade inflammation which is already present even in individuals with the virologically suppressed HIV. If these therapies can indeed eradicate all the infected cells, then the question arises as to whether these therapies can sufficiently diminish this underlying inflammation which are assumed to cause long-term effects on health.

## Conclusions

There is currently no vaccine for HIV. While highly active antiretroviral therapy has significantly reduced mortality, life expectancy among HIV-infected individuals remains lower than that of uninfected individuals. The increasing prevalence of non-communicable diseases (NCDs) as a contributor to this reduced life expectancy has highlighted the need for new strategies to improve long-term health outcomes. One emerging approach involves studying the gut microbiome and its dysbiosis to identify probiotic components that could promote gut health and overall wellness. The identification of biomarkers, such as NF-κB p65, as potential contributors to NCD development presents a novel approach to managing chronic, virally suppressed HIV-infected individuals. Could the routine estimation of NF-κB p65 levels be used to assess the risk of developing NCDs in these individuals? If so, what would be the cutoff value to indicate significant risk? Several questions remain unanswered as these strategies continue to be explored.
